# Bioinformatics analysis of miRNA and its associated genes to identify potential biomarkers of oral submucous fibrosis and oral malignancy

**DOI:** 10.1002/cnr2.1787

**Published:** 2023-01-28

**Authors:** Ezhuthachan Mithu Mohanan, Dhwani Jhala, Chandramani B. More, Amrutlal K. Patel, Chaitanya Joshi

**Affiliations:** ^1^ Gujarat Biotechnology Research Centre, Department of Science and Technology Government of Gujarat Gandhinagar Gujarat India; ^2^ Department of Oral Medicine & Radiology K.M. Shah Dental College and Hospital Vadodara Gujarat India

**Keywords:** Hub genes, miRDeep2, miRNA, miRNet, oral malignancy, oral submucous fibrosis

## Abstract

**Background:**

MicroRNAs are a group of non‐coding RNA that controls the gene expression. The interaction between miRNA and mRNA is thought to be dynamic. Oral cancer “The cancer of mouth” is quite prevailing in developing countries. miRNA has been found associated with oral cancer targeting tumor growth, cell proliferation, metastasis, invasion. The significant association of miRNA with genes could be used as a remarkable tool for diagnosis as well as prognostic analysis of oral cancer.

**Aim:**

The aim of the present study is to evaluate common upregulated and downregulated miRNAs in oral submucous fibrosis (OSMF) and oral malignancy (OM) patients that can be used as diagnostic biomarkers, and to find out their interactions with target genes to establish associated networks in cancer pathways.

**Methods and Results:**

Using miRDeep2 and DESeq analysis, the upregulated and downregulated miRNA in OSMF (Oral Submucous Fibrosis) and OM (Oral Malignancies) samples were compared to GEO (Gene Expression Omnibus) control dataset. There were 50 common downregulated miRNAs and 13 common upregulated miRNAs in OSMF and OM samples. miRNet analysis of common upregulated miRNA and common downregulated miRNA identified 1295 and 5954 genes, respectively connected with cancer pathways. From analysis of Hub genes, HRAS, STAT3, TP53, MYC, PTEN, CTNNB1, CCND1, JUN, VEGFA, KRAS were found associated with downregulated miRNA and VEGFA, TP53, MDM2, PTEN, MYC, ERBB2, CDKN1A, HSP90AA1, CCND1, AKTI were found associated with upregulated miRNA. The gene enrichment analysis of these hub genes were associated with cell communication, metabolic process, cell proliferation, and cellular component organization. Hub Genes linked with upregulated miRNA had an enrichment ratio of 11.828, whereas hub genes linked with downregulated miRNA had an enrichment ratio of 45.912.

**Conclusion:**

We identified common deregulated miRNAs between OSMF and OM patients, which were further analyzed to find out associations with the genes correlated to cancer pathways. The hub genes identified in this study were found to have a significant impact on tumor growth and carcinogenesis. Also, the enrichment of these genes has revealed that the genes are associated with cellular communication, metabolic processes and various biological regulation. These deregulated miRNAs can be used to make a panel of biomarkers to diagnose oral cancer from blood even before its onset.

## 1 INTRODUCTION

Oral cancer, commonly known as head and neck cancer, is a type of cancer that affects the lips, mouth, nasopharynx, and pharynx. It has a disproportionately high incidence in South Central Asia due to the prevalence of risk factors such as cigarette smoking, excessive alcohol intake, and consumption of areca nut. It is the world's sixth most frequent cancer and India's third most prevalent cancer.^1^ As per the report, there are nearly 77 000 new oral cancer cases and 52 000 fatalities per year in India.^2^ Furthermore, as compared to the Western countries, oral cancer is even more of a concern in India, since almost 70% of cases are identified in advanced stages, making the chances of cure extremely rare, with only a 20% survival rate.^3^

Oral cancer is sometimes preceded by other oral illnesses, such as oral submucous fibrosis (OSMF). OSMF is a precancerous condition characterized by ulceration and fibrosis in the sub mucosal tissues of the oral, oropharyngeal, and esophageal areas.^4^ In OSMF patients, the rate of malignant transformation might be as high as 30%.^5^, ^6^

At the post‐transcriptional stage, microRNAs play a crucial role in regulating protein expression affecting a variety of physiological functions. RNA biology research has found that small regulatory molecules known as miRNAs play a critical role in gene control across time. Many disorders, including malignancies, have been linked to the deregulation of these riboswitches.^7^ miRNAs can be released by cells, circulated in the bloodstream, or taken up by other cells.^8^, ^9^ Previous research has revealed that circulating miRNAs are associated with either RNA‐binding proteins or high‐density lipoproteins, or are encapsulated within extracellular vesicles (EVs).^9^, ^10^, ^11^ Researchers have identified the existence of cancer‐associated miRNAs in the circulatory system of oral cancer patients, as well as their abnormal expression.^12^, ^13^, ^14^, ^15^ The aberrant expression of microRNA causes many genes to malfunction, which can lead to various cancer progressions. According to studies MicroRNA is variably expressed in many malignancies, including oral submucous fibrosis and oral leukoplakia.^16^ Protein and gene interaction can be studied using bioinformatics and computational biology. Functional examination of these hub genes can assist researchers to figure out how they are linked to biological processes. The major goal of this study is to compare the deregulated miRNAs identified in oral malignancies and oral submucous fibrosis to uncover miRNA‐mRNA interactions related to cancer.

## 2 MATERIALS AND METHODS

### 2.1 Sample collection

Blood samples from 4 patients of OSMF (2 male, 2 female) and 3 patients of oral malignancy (2 male, 1 female) were collected from K. M. Shah Dental college and hospital, Sumandeep Vidyapeeth, Vadodara in 2019. The patients who are clinically and histopathologically diagnosed of having oral submucous fibrosis and who are older than 18 years were included as OSMF patients. Patients with history of cancer, radiotherapy and/or chemotherapy were excluded from OSMF patient category. For oral cancer samples, patients with clinical and histopathological diagnosis of oral malignancy (localized and metastatic) and older than 18 years were included. Patients with a previous history of radiotherapy and/or chemotherapy were excluded from the study. Prior approval from the Sumandeep Vidyapeeth Institutional Ethics Committee was taken and written informed consent was taken from each participant. A total of 2.5 mL blood of each person was collected directly in PAXgene® blood RNA tubes (BD Biosciences, USA, Cat. No. 762165). As per the manufacturer's protocol, the tubes were gently inverted 8 to 10 times, kept at room temperature for minimum 2 h and then stored at 4°C. The collected samples were brought to GBRC laboratory for further processing.

### 2.2 RNA isolation

Total RNA from blood samples was isolated as per the protocol given in the PAXgene Blood miRNA Kit Handbook (PreAnalytiX, Switzerland, Cat. No. 763134). After the collection, RNA samples were stored at −40°C until processed further. The concentration and purity of RNA was measured using Qubit 4 fluorometer (Thermo Fisher, USA) and Qiaxpert (Qiagen, USA), respectively.

### 2.3 Library preparation and sequencing

Library preparation was performed using Ion Total RNA‐Seq Kit v2 (Thermo Fisher, USA, Cat No. 4479789). The total RNA was first enriched for small RNA and then the library was constructed as per the manufacturer's protocol. Sequencing was carried out using 540 chip (Thermo Fisher, USA, Cat. No. A27765) on the Ion S5™ System (Thermo Fisher, USA).

### 2.4 Data sourcing for control

For control data, high throughput miRNA sequencing data were downloaded from NCBI GEO with accession ID GSE104440. The data has three sample sets, viz. Normal, Oral Leukoplakia, and Oral Squamous Cell Carcinoma. The control data (diagnosed to be healthy) which is of 20 pooled samples with ID GSM2800600 was used for the study.

### 2.5 miRNA annotation

The miRNA expression analysis was performed using the miRDeep2 software package (Version 2.0.1.3). The reads that passed quality control were mapped with the human reference genome hg38. Bowtie (version:2.4.4) was used to map the reads to the genome. The processed reads were then mapped using mapper.pl script with a three letter symbol ‘hsa’ for *Homo sapiens*. The outcome was non‐redundant reads with counts, source, and genomic location. miRDeep2 was used to annotate putative novel miRNA as well as used for validation of predictive miRNA from miRBase (version 22.0). For quantification of expression level of known miRNA, quantifier.pl script was used.^17^

### 2.6 Differential expression analysis

DESeq2 is used for modeling of raw counts, by using normalization factors. It is used for the estimation of gene wise dispersion. The hypothesis uses Wald test or likelihood ratio test. The total read count generated from the output of miRDeep2 was used as an input for DESeq2 package (version 1.32.0) and then further statistical analysis was conducted. DESeq2 likelihood ratio test is used for two conditions which include control versus disease samples. The Benjamini and Hochberg false discovery rate (FDR) was used for the adjustment of the resulting *p*‐value. miRNA was considered differentially expressed if log‐2‐fold change was >2 and *p*‐value adjusted is <0.05.^18^ Heatmap of deregulated miRNA in OSMF and OM compared to control was generated.

### 2.7 miRNA network analysis

An integrated platform linking miRNA tool—miRNet (version 2.0) was used for predicting the miRNA–gene interactions. The common upregulated and common downregulated miRNA of OSMF and OM samples were used as input for gene predictions. The miRNA–gene interaction data were collected from miRTarBase v8.0 using mirBase ID. The manual curation and validation of the predicted‐miRNA–gene interactions are produced from the miRNA target prediction programs of miRNet. For functional pathway enrichment, KEGG (Kyoto Encyclopedia of Genes and Genomes) was performed. The pathways generated were linked with genes interacting with miRNA.^19^ For the statistical analysis of miRNA‐gene interaction and finding the enriched pathways, minimum network connections was used as a filter. For protein–protein interaction (PPI) network formation, String database was used (version 11.0) (https://string-db.org/). PPI network analysis is a powerful tool for understanding biological responses. In the PPI network, a protein is defined as a node, and the interaction between two nodes is defined as an edge. The size of a node represents a degree: the larger the node, the larger the degree. The thickness of an edge indicates a correlation: the thicker the edge, the higher the correlation.^20^ The associated genes of upregulated and downregulated miRNAs were utilized as an input for the analysis of PPI networks with a confidence score of 0.4. Genes in the identified modules were placed into the STRING online database (http://string-db.org/). The pathway was investigated using KEGG.^21^ Cytoscape software was used to analyze the interactions between gene pairs (version 3.8.2).^22^ The purpose of Cytoscape, an open platform with many plugins, is to increase the capabilities of network analysis and visualization. Maximal clique centrality (MCC) was used to determine the top 10 hub nodes, and the Cytoscape MCODE plugin (version 2.0.0) was used to determine the core subnetworks with the most densely interconnected nodes.^23^ Hub genes are genes with a high correlation in candidate modules. The parameters set for the analysis using MCODE was score >3 number of node >5, degree cut‐off≥2, node score cut‐off ≥2, K‐core ≥2 and max depth = 100. The connectivity ranked at top 10%. The gene module membership >0.80 and gene trait significance >0.20 was set for the study of hub genes.

### 2.8 Functional and pathway enrichment analysis of hub genes

Web‐based Gene Set Analysis Tool kit (WebGeStalt) is a web tool used for the data retrieval, organization, visualization and statistical studies of functional enrichment. It is mainly used for the analysis of large gene sets. The graphical representation of data involves cellular components, biological processes, molecular functions and biological pathways. For this study, the FDR was set to be <0.05. The top 10 hub genes associated with upregulated and downregulated miRNA was used for the enrichment study using WebGeStalt. For the study of enrichment analysis, over representation analysis (ORA) method was used. It is a statistical method that determines genes from predefined sets which are present more than their expected value in a subset of data. The functional database used for the study was Gene Ontology (GO).^24^

## 3 RESULTS

### 3.1 Known and novel miRNA in OSMF and OM samples

The fastq files obtained after sequencing have been uploaded in NCBI SRA dataset (SRR18427233, SRR18427232, SRR18427231, SRR18427230, SRR18427229, SRR18427228, and SRR18427227).^25^ The RNA sequencing data from blood samples of 4 OSMF patients and 3 OM patients were processed in mirDeep2, the results of the same are shown in Table 1. The oral submucous fibrosis samples showed 513 ± 85.97 known miRNAs, whereas 296.75 ± 149.73 novel miRNAs. The oral malignancy samples had 175 ± 56.24 known miRNAs with only 4.33 ± 4.04 novel miRNAs. Novel miRNAs are those miRNAs which have not been reported in any of the miRNA databases (miRBase datasets). Thus, a low number of novel miRNA in case of oral malignancy is justified because it is studied more extensively than OSMF. As focus of this research is to find common signatures of deregulated miRNAs related to cancer pathways between different oral diseases, the novel miRNAs found here can be studied separately.

**TABLE 1:** miRDeep2 analysis of oral submucous fibrosis and oral malignancy.

Oral submucous fibrosis			Oral malignancy		
Sample ID	Known miRNA	Novel miRNA	Sample ID	Known miRNA	Novel miRNA
SRR18427230	458	169	SRR18427233	116	0
SRR18427229	619	303	SRR18427232	228	8
SRR18427228	545	505	SRR18427231	181	5
SRR18427227	430	210			

### 3.2 Identification of differentially expressed miRNAs

**FIGURE 1:**
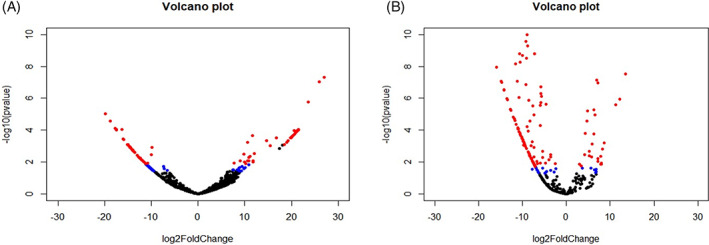
(A) DeSeq2 analysis of OSMF with GEO Control (GSE104440). (B) DeSeq2 analysis of OM with GEO Control (GSE104440). Red dots indicate significant genes with log2Foldchange > |2| and *p*_adj_ < 0.05.

**FIGURE 2:**
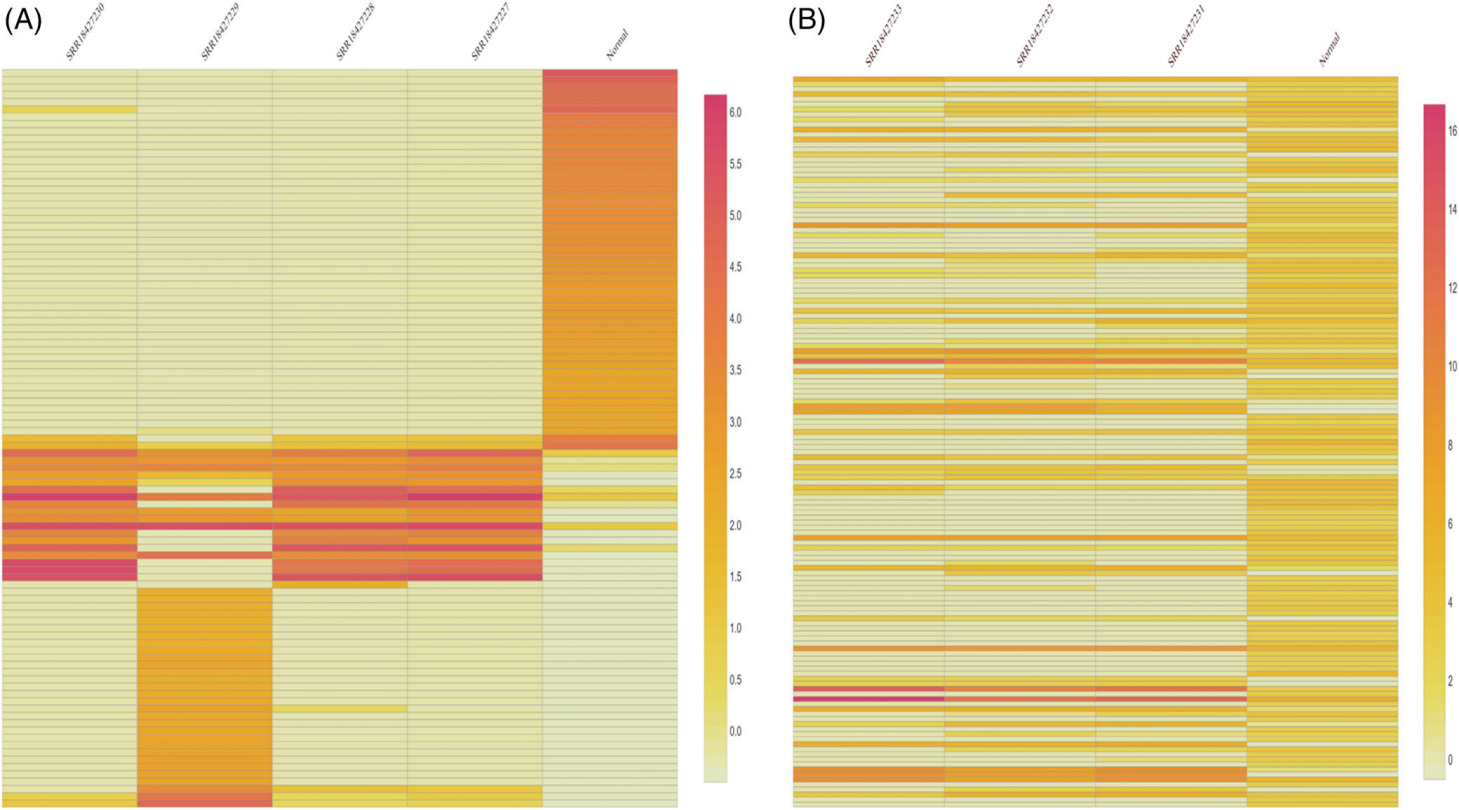
(A) Heat map generated of differentially expressed miRNA in OSMF compared with GEO control data (B) Heat map generated of differentially expressed miRNA of OM compared with GEO control data.

**FIGURE 3:**
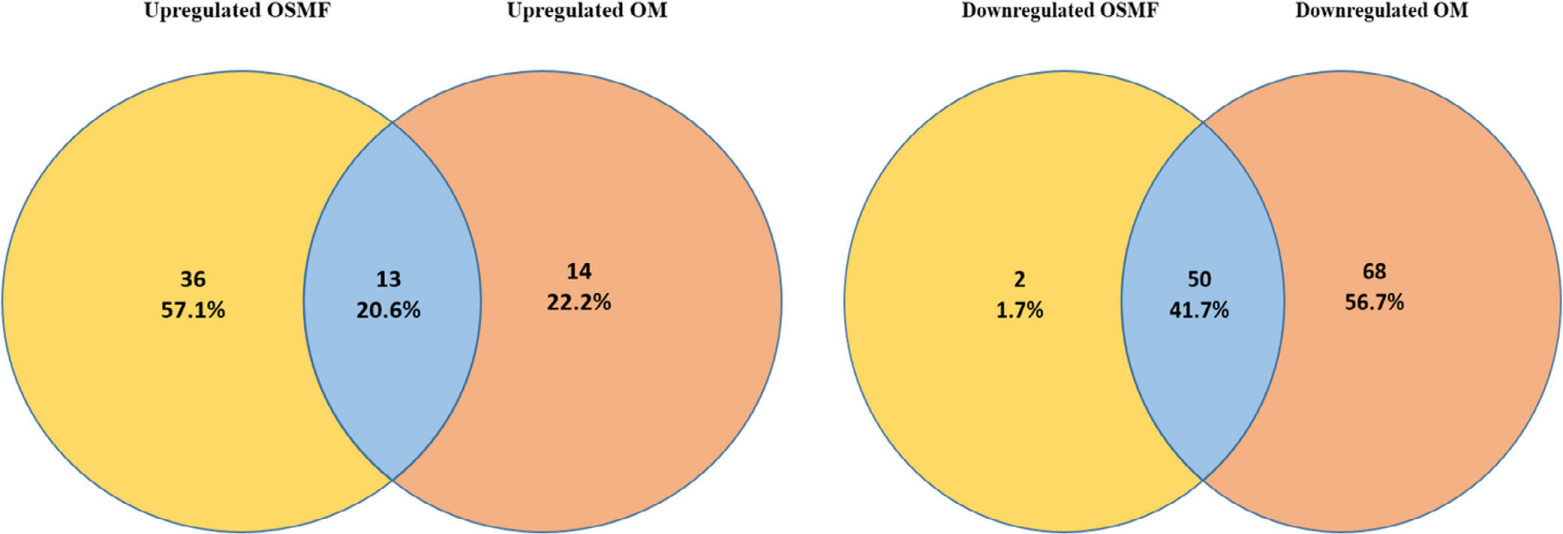
Venn diagram showing upregulated (A) and downregulated (B) miRNAs between OSMF and OM.

The known miRNA from both OSMF and OM samples were processed in DESeq2 to know differentially expressed, that is, upregulated or downregulated miRNA compared to miRNA profile of healthy individuals. DESeq2 detected differentially expressed miRNA (DEmiR) in OSMF and OM samples as shown in Figure 1. Upregulated and downregulated miRNAs are indicated by red dots in the figures. In OSMF samples, 49 miRNA were found to be upregulated and 52 were found to be downregulated when compared to GEO healthy control (Figure 1A), whereas in OM samples, 27 miRNA were found to be upregulated and 118 were found to be downregulated when compared to GEO healthy control (Figure 1B) (Table 2 and Table 3). The heat map for the OSMF and OM samples was generated. Each column indicates sample, while the color intensity indicates relative expression levels of deregulated miRNAs compared to the healthy control (Figure 2). When the downregulated and upregulated miRNAs of OSMF and OM samples were compared, it indicated that 50 miRNAs were commonly downregulated and 13 miRNAs were commonly upregulated. Venn diagram for the same is shown in Figure 3. The common miRNAs could be used to predict transformation of oral submucous fibrosis patients into oral cancer patients. These miRNAs were studied further in detail to know their target genes and related metabolic pathways.

**TABLE 2:** Top deregulated miRNA in OSMF.

Top five most downregulated miRNA in OSMF samples		
miRNA	log2FoldChange	padj
hsa‐miR‐22‐3p	−19.82432392	0.000950525
hsa‐miR‐25‐3p	−18.82143874	0.002096567
hsa‐miR‐103a‐3p	−17.7052872	0.002673139
hsa‐miR‐27b‐3p	−17.52156439	0.002673139
hsa‐miR‐143‐3p	−17.42806062	0.002673139

Top five most upregulated miRNA in OSMF samples		
miRNA	log2FoldChange	padj
hsa‐miR‐219b‐5p	26.97332362	1.83 E‐05
hsa‐miR‐323b‐5p	25.95278182	1.83 E‐05
hsa‐miR‐4433a‐5p	23.64140306	0.000232897
hsa‐miR‐4324	21.47570679	0.002673139
hsa‐miR‐1291	21.45857964	0.002673139

**TABLE 3:** Top deregulated miRNA in OM.

Top five most downregulated miRNA in OM samples		
miRNA	log2FoldChange	padj
hsa‐miR‐22‐3p	−15.84001293	3.38 E‐07
hsa‐miR‐25‐3p	−14.83710478	2.01 E‐06
hsa‐miR‐451a	−14.78074411	2.11 E‐06
hsa‐miR‐151a‐3p	−14.22748736	4.99 E‐06
hsa‐miR‐10b‐5p	−14.2107262	4.99 E‐06

Top five most upregulated miRNA in OM samples		
miRNA	log2FoldChange	padj
hsa‐miR‐92a‐2‐5p	13.49525728	8.04 E‐07
hsa‐miR‐19b‐1‐5p	12.2291664	1.52 E‐05
hsa‐miR‐19b‐2‐5p	12.2291664	1.52 E‐05
hsa‐miR‐7‐5p	11.20288297	2.86 E‐05
hsa‐miR‐1180‐5p	8.648993702	0.003077129

### 3.3 Prediction and analysis for target genes of miRNAs

**FIGURE 4:**
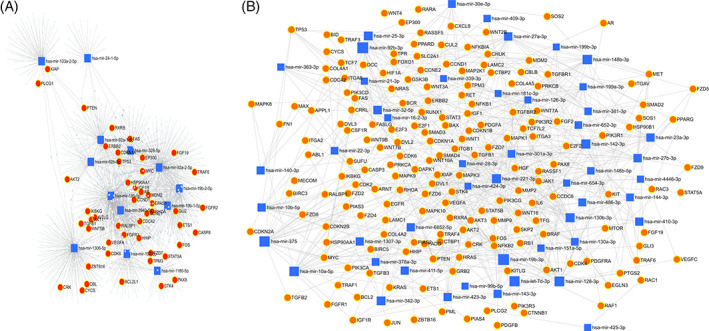
(A) Network analysis of common upregulated miRNA correlated to 47 target pathways in cancer with padj value 9 e‐5 (B) Network analysis of common downregulated miRNA correlated to 185 pathways in cancer with padj value 7.87 e‐19. The red circles indicate targeted genes in pathways of cancer, whereas the blue squares are common upregulated and downregulated miRNA.

Using pathway analysis, an in silico model of the functional effects of dysregulated miRNA was created in this study using the predicted gene targets. For each gene and miRNA node, the miRNet database offers several measurements of network connectivity and interactions, including node degree, or the number of nodes directly connected to a given node, and betweenness centrality, a more comprehensive analysis of network structure that counts the shortest paths through a node.^26^ The hypergeometric method was used to design a network with 1295 gene nodes and 1601 edges for commonly upregulated miRNA and 5954 gene nodes and 12 520 edges for commonly downregulated miRNA (Figure 4).^27^ The target hit was 47 with adj.p value of 0.00009 for common upregulated miRNA of OSMF and OM samples, while the target hit was 185 with adj.*p* value of 7.87 e−19 for common down‐regulated miRNA of OSMF and OM samples.

### 3.4 Analysis of hub genes

**FIGURE 5:**
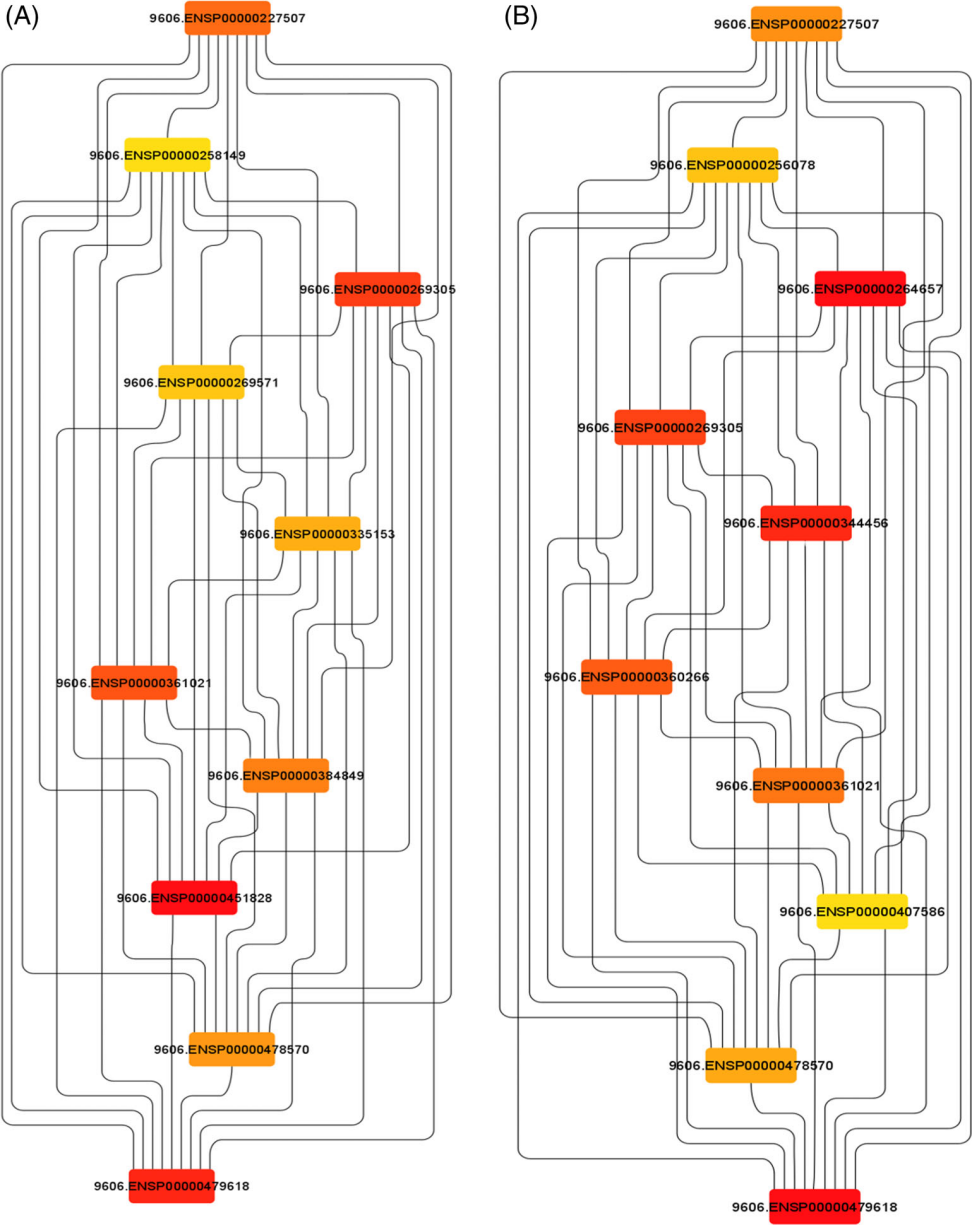
(A) Hub genes analyzed by CytoHubba associated with pathways of cancer obtained from common upregulated miRNA (B) Hub genes analyzed by CytoHubba from genes associated with pathways of cancer obtained from common downregulated miRNA.

**FIGURE 6:**
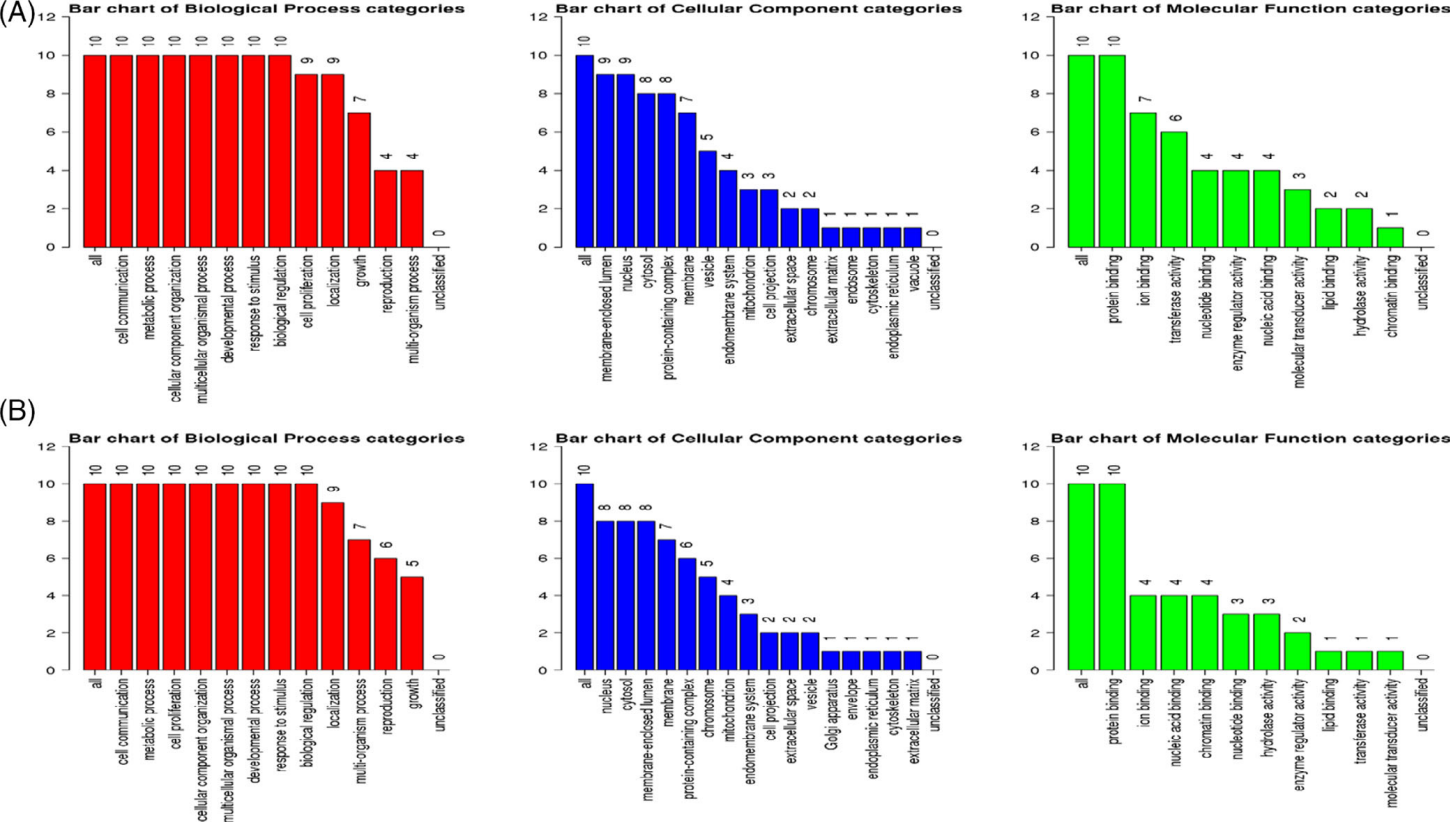
Gene annotation of Hub genes found from associated common upregulated (A) and downregulated (B) miRNA using WebGeStalt.

For the analysis of Hub genes, a local based MCC (maximal clique centrality) score method was used because it captures more essential proteins in the top ranked list and also discovers feature nodes. The main rational in using MCC is that essential proteins are clustered in protein–protein interaction.^28^ From analysis of Hub genes, *HRAS*, *STAT3*, *TP53*, *MYC*, *PTEN*, *CTNNB1*, *CCND1*, *JUN*, *VEGFA*, and *KRAS* were found to be associated with common downregulated miRNAs and *VEGFA*, *TP53*, *MDM2*, *PTEN*, *MYC*, *ERBB2*, *CDKN1A*, *HSP90AA1*, *CCND1*, and *AKTI* were found to be associated with common upregulated miRNAs (Figure 5). For gene enrichment analysis, WebGeStalt was used (Figure 6). When gene enrichment for 10 hub genes associated with upregulated miRNAs was performed, it was found that all 10 genes were found to be enriched in cell communication, metabolic processes, cell organization and protein binding. Nine genes were found to be present in membrane‐bound nucleus. Hub genes linked with upregulated miRNAs had an enrichment ratio of 11.828. When gene enrichment for 10 hub genes associated with downregulated miRNAs was performed, it was found that all 10 genes were found to be enriched in cell communication, metabolic processes, and cell proliferation. Eight genes were found in nucleus and cytosol and 10 genes were involved with protein binding. Hub genes linked with downregulated miRNAs had an enrichment ratio of 45.912.

## 4 DISCUSSION

Major etiological factors that induce oral squamous cell carcinoma are excessive intake of betel, alcohol consumption, poor diet, immune deficiency, and so on. Late diagnosis of cancer leads to metastasis in lymph nodes, thus jeopardizing clinical treatment. By a report, one‐third of oral submucous fibrosis has the capacity to develop into slow‐growing squamous cell carcinoma.^29^ India has a high consumption of beetle nut areca, the case–control study showed that among 100 oral cancer patients, 40 patients showed features of oral submucous cell fibrosis, thus validating the malignant potential of OSMF.^30^ Similarly, in another study of 119 OSMF patients in Central India, the malignant transformation was found 4.2%.^31^ Besides the transformation of OSMF to OSCC, there are also studies that show miRNA is involved in the regulation of OSMF transfer to OSCC.^32^, ^33^, ^34^ Additionally, recent studies have demonstrated that ruptured cells release miRs in the circulatory system including body fluids such as blood and urine, etc. The discovery of the circulating miRs in blood serum has emerged as an early biomarker in cancer.^33^ Circulating miRs have become a potential molecular biomarker for early diagnosis of cancer due to their abundance in body fluids.^35^ There are studies where miRNA expression was analyzed from the blood of OSMF patients.^36^, ^37^, ^38^, ^39^ The identified miRNAs and the target genes have a major impact on understanding the molecular mechanism and can be helpful to identify the targets which could be used for diagnosis. In this study, we found that hsa‐miR‐219b‐5p was the most upregulated miRNA in the OSMF samples, whereas hsa‐miR‐92a‐2‐5p was the most upregulated miRNA in the OM samples. No study has been reported pertaining to regulation by hsa‐miR‐219‐5p in oral submucosa fibrosis cancer, but since OSMF is a pre‐cancer stage, overexpression of hsa‐miR‐219b‐5p may be to prevent metastasis as reported in other cancerous studies.^40^ According to certain studies, hsa‐miR‐92a‐2‐5p is increased in cancers such as non‐small‐cell lung cancer (NSCLC).^41^ Interestingly, the most down regulated miRNA in both the OSMF and OM samples was hsa‐miR‐22‐3p. Overexpression of the hsa‐miR‐22‐3p causes growth suppression,^42^ and this is observed to be downregulated in both OSMF and OM. A network analysis using miRNet helped to understand the regulatory mechanism of miRNA by its interaction with targeted genes. There were many diseases found to be associated with miRNA network analysis of common upregulated and common down regulated miRNA. This study focused on understanding those miRNA‐gene interactions which were enriched in pathways in cancer. From the genes associated with pathways in cancer Hub genes were analyzed using the MCC method.^43^, ^44^ Analysis of Hub genes identified *HRAS*, *STAT3*, *TP53*, *MYC*, *PTEN*, *CTNNB1*, *CCND1*, *JUN*, *VEGFA*, and *KRAS* associated with downregulated miRNA and *VEGFA*, *TP53*, *MDM2*, *PTEN*, *MYC*, *ERBB2*, *CDKN1A*, *HSP90AA1*, *CCND1*, and *AKTI* associated with upregulated miRNAs. These findings are supported by previous studies. Reports suggest that *MYC i*s found to be associated with oral cancer progression. Over‐expression of *MYC* can cause cancer cell proliferation.^45^ *JAK2/STAT3* is a well‐known oncogenic pathway that leads to a variety of solid malignancies, including oral squamous cell carcinoma. Anti‐apoptosis, angiogenesis, and migration are all aided by *STAT3*. According to some research, *STAT3* is activated in 70% of solid tumors.^46^, ^47^ In the presence of Wnt signaling, *CTNNB1* encodes catenin, which plays a key role in cell–cell adhesion adaptor protein as well as transcriptional coregulation.^48^ Upregulation of *CTNNB1* and *CCND1* may also increase cancer cell invasion, according to certain reports.^49^ Activated c‐Jun may play a key role in cancer development and carcinogenesis.^50^ Dysregulation of *KRAS* leads to various hallmarks of cancer like proliferation, metabolic reprogramming, anti‐apoptosis, and metastasis.^51^ There are reports suggesting ras as protooncogenes, from which *HRAS* appears as highly prevalent in OSCC.^52^

Vascular endothelial growth factor (*VEGF‐A*) is a key angiogenesis stimulator. There are studies that report *VEGF‐A* regulating tumor progression. Cases of association of *VEGF‐A* isoforms with oral squamous cell carcinoma have been reported. Patel et al. reported that the transcription level of *VEGF‐A* gene is high in oral carcinoma tissue compared to normal tissue.^53^

In conclusion, the bioinformatics analysis of reported biomarkers may be used to predict disease progression, if future analysis with larger sample size shows consistent results. miRNA analyzed using miRDeep2 is associated with the genes which have mostly correlated with the cancer pathway. Also, the enrichment of these genes has revealed that the genes are associated with cellular communication, metabolic processes and various biological regulation. Thus, dysregulation of these genes may have a role in tumor progression. Although our study is limited by sample size, it proves that miRNAs play critical roles in both OSMF and OSCC. The analysis of hub genes and its interaction can serve as candidate biomarkers to target potential diagnosis. In future, in vitro verification of the DEmiRs and relevant genes can be done for concrete validation. Also, the study can be performed on a larger cohort for statistical analysis.

## AUTHOR CONTRIBUTIONS

**Ezhuthachan Mithu Mohanan:** Formal analysis (equal); investigation (equal); writing – original draft (equal). **Dhwani Jhala:** Investigation (equal); writing – original draft (equal); writing – review and editing (equal). **Chandramani More:** Resources (equal). **Amrutlal Patel:** Methodology (equal); writing – review and editing (equal). **Chaitanya Joshi:** Conceptualization (equal); methodology (equal).

## FUNDING INFORMATION

This work was supported by the Department of Science and Technology (DST), Government of Gujarat.

## CONFLICT OF INTEREST STATEMENT

The authors declare no competing interests.

## ETHICS STATEMENT

This study was approved by the Sumandeep Vidyapeeth Institutional Ethics Committee and informed consent was taken from each participant.

## Data Availability

All data generated or analyzed during this study are available in online public repositories, within the article.
